# Dietary *Bacillus toyonensis* BCT-7112^T^ Supplementation Influences Performance, Egg Quality, Ammonia Emission, and Cecal Microbiome in Laying Ducks

**DOI:** 10.3390/vetsci12030259

**Published:** 2025-03-10

**Authors:** Tossaporn Incharoen, Rangsun Charoensook, Wandee Tartrakoon, Sonthaya Numthuam, Yutthana Sunanta, Guillermo Jimenez, Juan J. Loor

**Affiliations:** 1 Division of Animal Science and Feed Technology, Department of Agricultural Sciences, Faculty of Agriculture, Natural Resources and Environment, Naresuan University, Phitsanulok 65000, Thailand; tossaporni@nu.ac.th (T.I.); wandeeta@nu.ac.th (W.T.); sonthayan@nu.ac.th (S.N.); 2 Center of Excellence in Nonlinear Analysis and Optimization, Faculty of Science, Naresuan University, Phitsanulok 65000, Thailand; 3 Faculty of Animal Science and Technology, Maejo University, Chiang-Mai 50290, Thailand; yutthana_s@mju.ac.th; 4 Rubinum S.A., 08191 Rubí, Catalonia, Spain; g.jimenez@groupandersen.com; 5 Division of Nutritional Sciences, Department of Animal Sciences, University of Illinois, Urbana, IL 61801, USA; jloor@illinois.edu

**Keywords:** ammonia emission, *Bacillus toyonesis*, egg performance, gut microbiome, laying ducks

## Abstract

This study evaluated the effects of *Bacillus toyonensis* BCT-7112^T^ on laying duck performance, egg quality, ammonia emission, and cecal microbiota. Two hundred twenty 32-week-old Khaki Campbell ducks were assigned to four dietary treatments (0, 200, 500, or 1000 ppm Toyocerin^®^ 10^9^ premixture, containing 1 × 10⁹ *B. toyonensis* BCT-7112^T^ CFU/g) with five replicate pens of eleven birds each. Data on productivity, egg quality, and ammonia emissions were collected weekly, and cecal microbiota were analyzed at 44 weeks of age. Supplementation with 1000 ppm *B. toyonensis* BCT-7112^T^ significantly increased average egg weight (*p* < 0.001) and eggshell thickness (*p* = 0.007). Egg mass also improved at higher supplementation levels (*p* < 0.05), though feed intake, feed conversion, daily egg production, and most egg quality parameters were unaffected (*p* > 0.05). Ammonia emissions in litter decreased significantly (*p* < 0.05) with probiotic inclusion. Cecal microbiota analysis revealed higher diversity in ducks fed 1000 ppm, with a notable shift in predominant phyla from Bacteroidetes (35.12%) and Firmicutes (34.93%) in the controls to Bacteroidetes (40.52%), Firmicutes (34.08%), and Deferribacteres (9.54%) in the treated ducks. The findings suggest that 1000 ppm *B. toyonensis* BCT-7112^T^ enhances egg production, eggshell quality, and microbial diversity while reducing ammonia emissions in laying duck systems.

## Simple Summary

Duck egg production is crucial to Thailand’s poultry industry, and supplying eggs is crucial to Thai and Chinese cuisine. While traditional duck farming occurs outdoors on rice fields, modern intensive farming prioritizes productivity and disease control, often relying on antibiotics. However, growing health and environmental concerns necessitate alternatives like probiotics. This study explored the effects of *Bacillus toyonensis* BCT-7112^T^, a non-toxigenic, spore-forming bacterium, on Khaki Campbell laying ducks. The results show improved egg weight and shell thickness, reduced ammonia emissions, and increased cecal microbiota diversity. Our findings suggest *B. toyonensis* BCT-7112^T^ enhances egg production, gut health, and microbial diversity, offering a sustainable feed additive for intensive duck farming.

## 1. Introduction

Duck egg production is one of Thailand’s most important commercial poultry product sectors. Eggs from ducks are a popular ingredient in many recipes for both Thai and Chinese cuisines. In Thailand, ducks are traditionally raised outdoors on harvested rice fields [1]. However, in order to enhance productivity and prevent avian influenza infection from wild birds, poultry producers are increasingly turning to indoor industrial farming [2,3]. The way of raising laying ducks is changing from free-range on harvest paddy fields to intensive or semi-intensive systems. Because they are raised in a setting with high density and high production pressure, poultry may be exposed to various stressors and pathogen infections, which could affect their health and productivity. As a result, feeding antibiotic growth promoters (AGPs) is a frequent practice in the poultry production industry. However, the long-term usage of AGPs may result in bacterial resistance and antibiotic residues in poultry products and the environment [3,4]. Furthermore, with high-density rearing in intensive systems, ammonia damage is expected to be a significant issue in poultry production [5].

Since the ban on AGPs in the livestock industry, using probiotics as feed supplements in animal agriculture has grown dramatically over the last decade. Because of their ability to form spores, certain non-pathogenic Gram-positive lactic acid bacteria (LAB), such as Bacillus sp., are appealing for use as a probiotic additive in livestock feed [6,7]. *Bacillus toyonensis* BCT-7112^T^ (previously known as *Bacillus cereus* var. *toyoi*) is a non-toxigenic, Gram-positive, facultatively anaerobic, spore-forming bacterium in the Bacillus cereus group. It has been utilized since 1975 as a feed additive for livestock and aquaculture in various nations worldwide [3,8]. Several studies reported that *Bacillus toyonensis* improves performance, immune stimulation, intestinal health, and nutrient absorption as well as reducing enteric infections, decreasing ammonia emission, and increasing the variety of microbial communities in the gastrointestinal tract of several target species, including rabbits [9], swine [10,11], ruminants [12,13], chickens [14], quails [15], turkeys [16,17], meat-type duck [3], and aquatic animals [18,19]. The effect of *Bacillus toyonensis* on laying ducks, on the other hand, has not been investigated.

The productive performance, egg quality, gut health, and ammonia emissions of ducks are all linked to their intestinal microflora [5]. Furthermore, the gut microbiota promotes intestinal barrier integrity, stimulates more commensals, and excludes pathogens to protect the host’s health, nutritional metabolism, and absorption, development of the immune system, and infection defense [3,20,21]. The conventional methods of microbial identification are frequently insufficient for identifying a large number of microorganisms present in habitats with microbial diversity. As a result of the rapid advancement of biotechnology, molecular identification based on genomic sequencing has garnered broad interest. In poultry, 16S rRNA gene sequencing is commonly applied in investigations of microbial diversity and community composition in the gut [3,5]. Thus, the objectives of this study were to investigate the effect of probiotic supplementation on performance, egg quality, ammonia emission, and cecal microbiota in laying ducks.

## 2. Materials and Methods

### 2.1. Ethics Statement

This investigation was conducted at the Poultry Nutrition and Feed Testing Unit affiliated with the Faculty of Agriculture, Natural Resources, and Environment at Naresuan University, Phitsanulok, Thailand. All animal procedures employed in the current research were approved and regulated by the Naresuan University Agricultural Animal Care and Use Committee (NUAACUC; reference number 61 01 009), following the Ethical Principles and Guidelines for the Use of Animals, National Research Council of Thailand.

### 2.2. Research Design and Animal Information

Three hundred 18-week-old Khaki Campbell laying ducks were purchased from a commercial layer duck farm (Charoenrat Farm, Phitsanulok province, Thailand). All birds were maintained in a temperature-controlled house using an evaporative system. They were reared in a litter and a slatted floor system equipped with a water bath and nest. The total number of eggs laid from each pen was recorded daily to determine the egg production percentage. Feed and drinking water were provided ad libitum during the pre-experimental period. At 32 weeks old, 220 ducks with laying and body weight uniformity were selected and assigned to four dietary treatments with five replicates per treatment and 11 birds per replicate. The experimental diets were a basal control (Table 1) supplemented with 0, 200, 500, or 1000 ppm (mg/kg) Toyocerin^®^ 10^9^ premixture (containing 1 × 10^9^ *Bacillus toyonensis* BCT-7112^T^ CFU/g, Rubinum SA, Barcelona, Spain). The dosage levels were based on our previous study and manufacturer recommendations [3]. The basal diet with 18% crude protein level (Table 1) was formulated to satisfy the nutritional requirements for Khaki Campbell laying ducks, according to Likittrakulwong et al. [1]. Each dietary group provided feed and drinking water for ad libitum intake. The photoperiod was set at 17L: 7D through the experimental period (32 to 44 weeks old).

### 2.3. Data Collection for Laying Performance and Quality

Egg production and weight were recorded daily from each pen, including intact, dirty, and broken eggs. The average daily egg production was calculated based on weekly results as the following formula: [total number of eggs per pen/(number of hens per pen × days)] × 100. The total egg weight of each pen was divided by the total number of eggs per pen and expressed as the average egg weight. Egg mass multiplied by average egg weight by average daily egg production percentage. Feed intake was obtained by deducting the total feed weight offered during the week by the remaining feed weight. Average daily feed intake was modified for mortality and was calculated using the following equation: [total feed intake per pen/(number of hens per pen × days)]. The feed conversion ratio was calculated by dividing the average daily feed intake by egg mass.

An egg from each pen was collected weekly to analyze the egg quality during the 12-week feeding period as follows: yolk ratio, albumen ratio, eggshell ratio, albumen height, eggshell thickness, yolk color, and Haugh units. Dirty and cracked eggs were sorted out and excluded from analysis. After the breakout, eggshells, albumen, and yolk were separated and weighed using a digital precision scale. The ratio of eggshell, albumen, and yolk was calculated based on the weight of the whole egg. Yolk color, albumen height, and the Haugh unit were measured by an egg multitester (EMT-7300, Robotmation Co., Ltd., Tokyo, Japan). The shell thickness was measured at the sharp, blunt ends and equator after removing the shell membranes using a digital micrometer (MW200-01DBL, Moore & Wright Co., Ltd., Sheffield, UK).

### 2.4. Measurement of Ammonia Emission in Litter

The ammonia emission was measured at the litter level during the whole feeding trial (12-week experimental period), according to the modified method of Yamauchi et al. [23]. Twelve samples of fresh litter (rice hull) were collected weekly from 12 surface locations within each pen using a sampling spoon, and each one was transferred to a vinyl bag. Thus, a total of 60 litter samples were obtained per treatment every week (12 samples/pen × 5 pens/treatment, *n* = 60). From each litter sample, a total of 100 g was transferred to a tight container with a cap and a gap sealed with paraffin wax. Each container was placed in an incubator at 37 °C for one hour before being allowed to cool to room temperature for 30 min. A portable gas detector was used to measure the ammonia emission in each litter sample (Crowcon Portable Gas Detection; Crowcon Detection Instruments, Oxfordshire, UK). The gas collector tube was inserted into the air sampling hole, and the maximum ammonia emission values were recorded in ppm units. The ammonia emission was expressed as the average value of the 60 litter samples collected per treatment.

### 2.5. DNA Extraction and PCR Amplification

At the end of the feeding trial, five ducks from the control and treatment groups that showed the best laying performances were randomly chosen (one duck per replicate). The ducks were individually euthanized for cecal content collection and immediately transferred to a sterile 1.5 mL microcentrifuge tube and maintained at −80 °C until DNA extraction [3]. DNA was isolated from 200 mg of cecal contents from each duck using a QIAamp DNA Stool Mini Kit (Qiagen, Valencia, CA, USA) with modifications to improve DNA concentration. Isolated DNA concentrations and agarose gel electrophoresis were measured by spectrophotometry (NanoDrop 2000, Thermo Science, Wilmington, DE, USA) at OD A260/A280 ratio >1.8. The extracted DNA was normalized to a 50 ng/mL concentration and stored at −20 °C. The diluted DNA was then used as a template for the PCR amplification of the bacterial 16S rRNA genes, and the V3–V4 variable regions were amplified with specific primers for bacterial diversity analysis [3]. Equal amounts of purified amplicons were individualized for next-generation sequencing (NGS).

### 2.6. Determination of Cecal Microbiome by Next-Generation Sequencing (NGS)

According to the manufacturer’s instructions, the sequencing library and barcode were constructed using the TruSeq^®^ DNA PCR-sample-free preparation kit (Illumina, San Diego, CA, USA). The sequencing was conducted on the Illumina MiSeq platform, which produced 250 bp paired-end readings. Paired-end reads were assigned to samples using their unique barcodes and then truncated by removing the barcode and primer sequences. Paired-end reads were merged using FLASH (V1.2.7), a fast and accurate analysis tool designed to merge paired-end reads when at least some of them overlap with reads generated from the opposite end of the same DNA fragment, and the splicing sequences were referred to as raw tags. Specific filtering conditions were applied to the raw tags using the Qiime (V1.9.1) (http://qiime.org/scripts/split_libraries_fastq.html; accessed on 8 March 2022) quality-controlled process to obtain high-quality clean tags. The tags were compared against the reference database (Gold database) using the UCHIME algorithm (https://drive5.com/usearch/manual/chimeras.html; accessed on 8 March 2022) to detect and remove chimera sequences. The remaining sequences were considered effective clean reads.

All clean reads cluster analyses were performed by the Uparse software (Uparse v7.0.1001, http://drive5.com/uparse/; accessed on 8 March 2022) using all the effective tags. Sequences with ≥97% similarity were assigned to the same Operation Taxonomic Unit (OTUs) and the highest frequency sequence was selected as the representative for each OTU sequence. The Mothur software was used for each representative sequence to annotate species at each taxonomic rank (kingdom, phylum, class, order, family, genus, species) using the SILVA Database’s SSUrRNA database. The phylogenetic relationship of all OTU representative sequences was obtained using MUSCLE version 3.8.31 (http://www.drive5.com/muscle/; accessed on 8 March 2022), which can compare multiple sequences rapidly. The amount of OTU information was normalized using a standard sequence number corresponding to the sample with the fewest sequences. Lastly, alpha and beta diversity analyses were performed using the output normalized data.

Alpha diversity was applied to analyze the complexity of biodiversity for a sample through six indices, including observed species, Chao1, Shannon, Simpson, ACE, and good coverage. All these indices in our samples were calculated with QIIME version 1.7.0 and displayed with R software version 2.15.3. The unweighted UniFrac distance matrix was calculated for beta diversity metrics and the unweighted pair-group method with arithmetic means (UPGMA) cluster trees was applied using QIIME version 1.9.1. Principal component analysis, principal coordinate analysis (PCoA), and nonmetric multidimensional scaling figures were generated using R scripts [3].

### 2.7. Statistical Analysis

Egg performance, egg quality, and ammonia emissions from litter were analyzed using a one-way analysis of variance (ANOVA) with the Statistical Package for the Social Sciences (SPSS), version 17.0 (SPSS Inc., Chicago, IL, USA). The results are presented as group means along with the pooled standard error of the mean (SEM). Treatment differences were determined using Duncan’s multiple range test, with statistical significance set at *p* < 0.05.

## 3. Results

### 3.1. Laying Productivity and Egg Quality

The effects of different levels of dietary *Bacillus toyonensis* BCT-7112^T^ on the laying productivity and egg quality of laying ducks are reported in Table 2. Increasing dietary *Bacillus toyonensis* BCT-7112^T^ levels led to a tendency for egg mass to be higher compared with the control group, and responses were highest (*p* < 0.05) with 1000 ppm *Bacillus toyonensis* BCT-7112^T^. Compared to the control-fed group, all laying ducks *fed Bacillus toyonensis* BCT-7112^T^ had a significant improvement (*p* < 0.001) in average egg weight. However, in terms of the average daily egg production, feed conversion ratio, and feed intake, there were no significant differences (*p* > 0.05) among dietary treatments. Feeding *Bacillus toyonensis* BCT-7112^T^ increased eggshell thickness significantly (*p* = 0.007) compared to the control group. In contrast, no significant changes were detected for the yolk-to-albumen ratio, eggshell-to-albumen ratio, albumen height, Haugh units, or yolk color in response to *Bacillus toyonensis* BCT-7112^T^ compared to the control diet.

### 3.2. Quantity of Ammonia Emission in Litter

**Figure 1:**
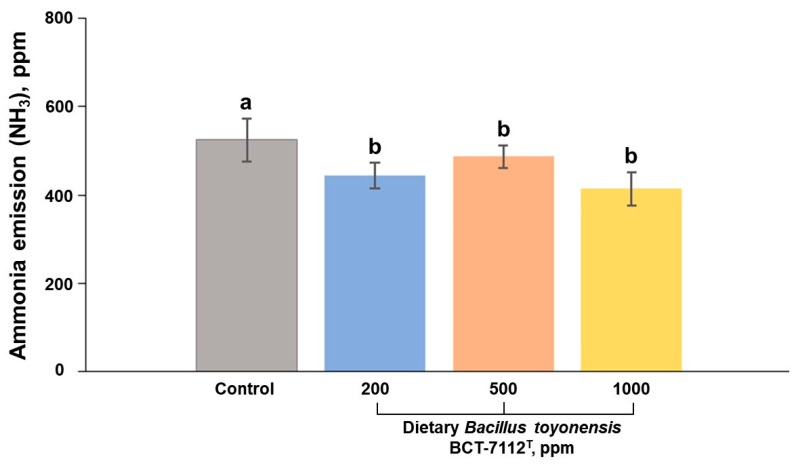
Average values of ammonia (NH_3_) emission in the litter (ppm) of laying ducks fed dietary *Bacillus toyonensis* BCT-7112^T^ (200, 500, 1000 ppm) from 32 to 44 weeks of age compared to the control group (*n* = 60 per group). Error bars represent the standard error of the mean (SEM = 10.57). Different superscripts letters indicate significant differences (*p* < 0.05).

The ammonia emission (NH_3_) in litter responses is depicted in Figure 1. Compared to the control group, all groups supplemented with *Bacillus toyonensis* BCT-7112^T^ had significantly reduced ammonia emission in the litter (*p* < 0.05).

### 3.3. Analysis of Microbial Communities in Laying Duck Cecum

**Figure 2:**
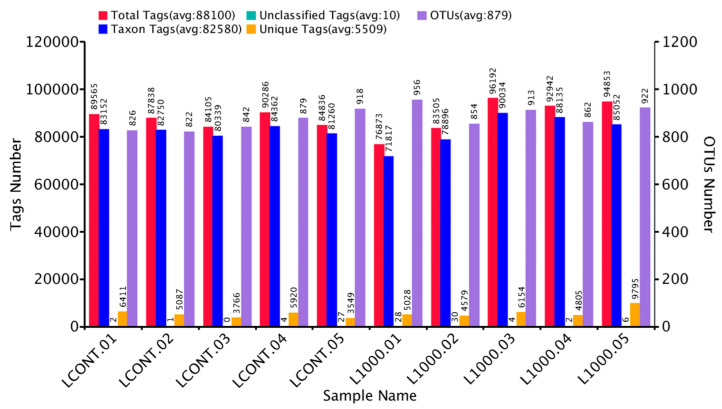
Summarization of the tags and OTU number of each sample in control (LCONT) and 1000 ppm (L1000) groups.

**Figure 3:**
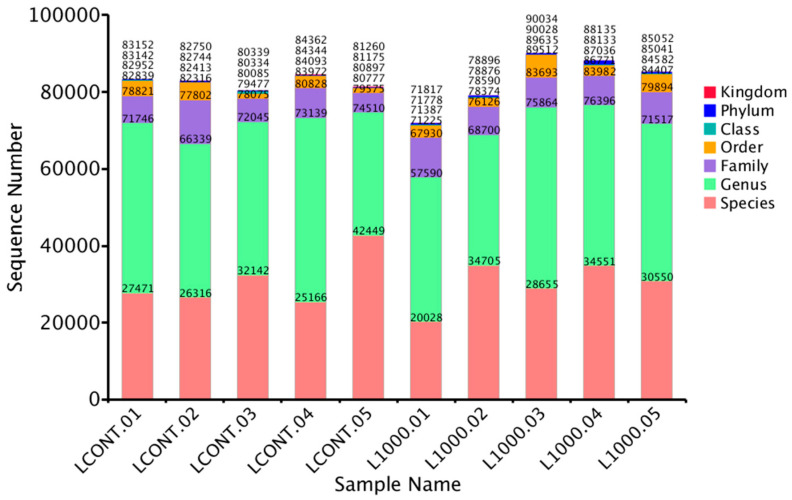
Summarization of the classified sequence and sequence number of each sample in the control (LCONT) and 1000 ppm (L1000) groups.

The gut microbiome analysis in this study was performed in the control group and the 1000 ppm *Bacillus toyonensis* BCT-7112^T^ group, which had the best laying performance among the supplemented groups. Basic information from various samples, such as effective tag data, low-frequency tag data, and tags annotation data, was collected to construct OTUs (Figure 2). To examine the species diversity in each sample, all effective tags were grouped into OTUs based on 97% DNA sequence similarity. The summary of classified sequence and sequence number for each sample are reported in Figure 3. Six different alpha diversity indices, including observed species, Shannon, Simpson Chao1, ACE, and Good’s coverage, were used to access the sequence richness of the sample and analyze diversity (Table 3).

**Figure 4:**
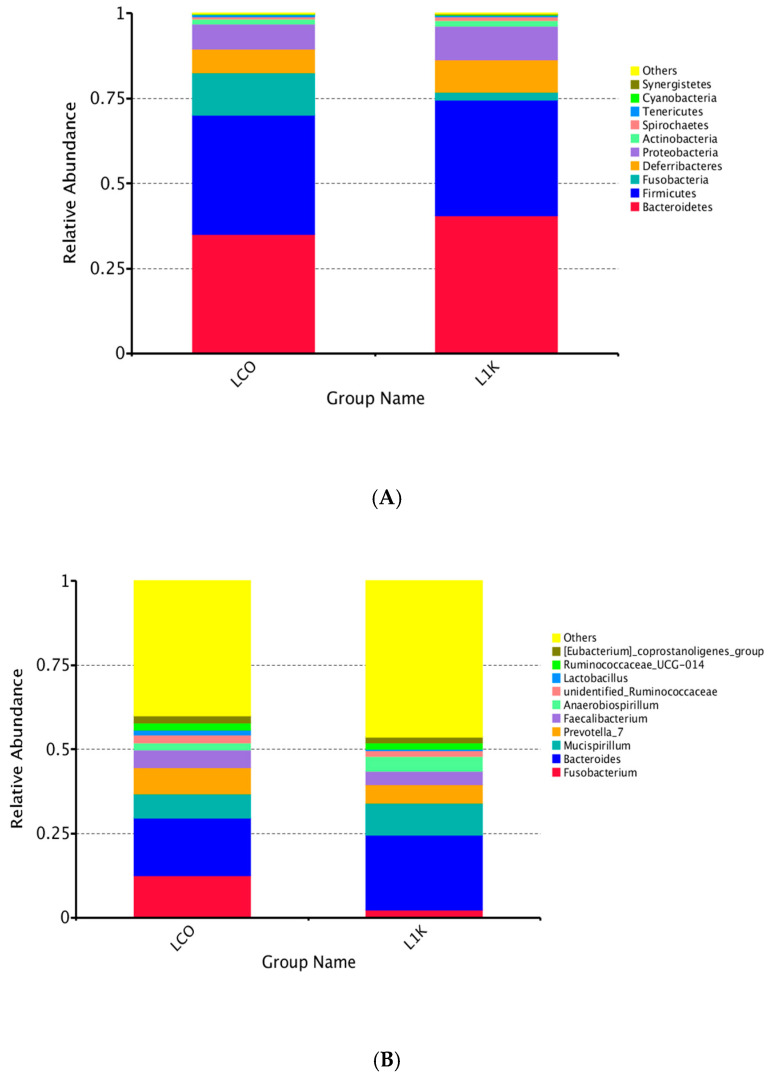
Relative abundance of cecal bacteria in laying ducks at the phylum (**A**) and genus (**B**) levels in the control (LCO) and 1000 ppm (L1K) groups.

The microbial population in the control group was dominated by the following phyla: Bacteroidetes (35.12%), Firmicutes (34.93%), Fusobacteria (7.38%), Proteobacteria (6.72%), and Deferribacteres (7.05%). In contrast, Bacteroidetes (40.52%), Firmicutes (34.08%), Proteobacteria (9.90%), Deferribacteres (9.54%), and Fusobacteria (2.24%) were the predominant phyla of the cecal microbiota in the 1000 ppm group (Figure 4).

**Figure 5:**
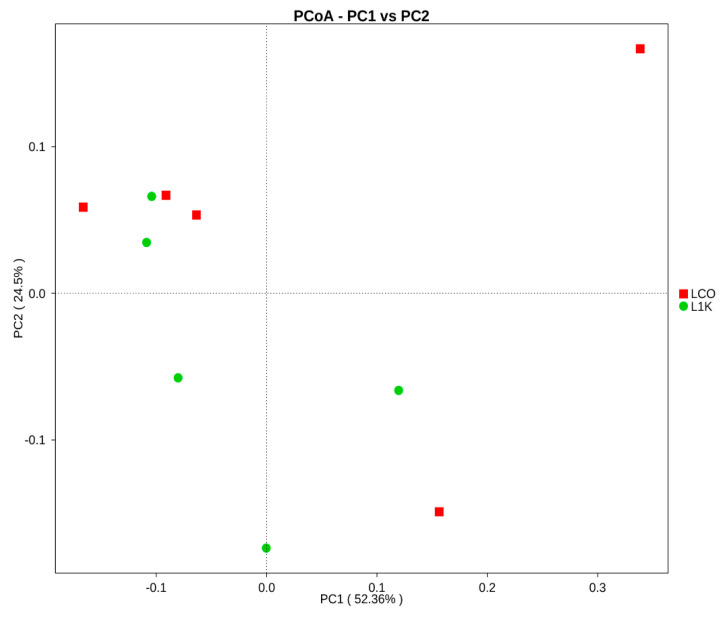
Principal component analysis (PCA) of the microbial community of the laying ducks in the control (LCO) and 1000 ppm (L1K) groups.

The top genera in the control group were *Bacteroides* (17.17%), *Fusobacterium* (12.42%), *Prevotella* 7 (12.42%), *Mucispirillum* (7.05%), *Faecalibacterium* (5.25%), and *Anaerobiospirillum* (2.27%). In contrast, *Bacteroides* (22.23%), *Mucispirllum* (9.54%), *Prevotella 7* (5.55%), *Anaerobiospirillum* (4.43%), *Faecalibacterium* (3.83%), and *Fusobacterium* (2.24%) were predominant in the microbiota from the 1000 ppm group (Figure 5). The explicit comparison of microbial communities according to their composition is known as beta diversity. In order to analyze the microbial communities between each set of community samples, the beta diversity evaluates their differences. The microbial communities in the cecum were visually depicted through a principal component analysis (PCA) (Figure 5). These data suggested that the gut microbiome may have changed due to dietary and environmental stressors, with the group fed 1000 ppm *Bacillus toyonensis* BCT-7112^T^ having a greater microbial diversity compared to the control.

**Figure 6:**
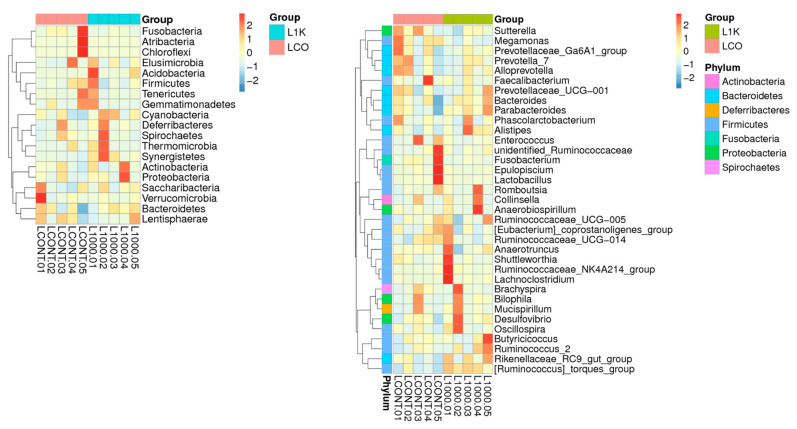
Heatmap analysis of 35 most abundant phyla and genera of each bird was carried out in the control (LCON.01–LCON.05) group with basal diet and 1000 ppm (L1000.01–1000.05 ppm) group with the B. toyonensis BCT-7112^T^ supplement. Notes: Plots by sample name on the X-axis and the Y-axis represent the phylum. The absolute value of ‘z’ represents the distance between the raw score and the mean of the standard deviation. ‘z’ is negative when the raw score is below the mean, and vice-versa (rank from −2 to 2).

The R software was used to cluster and plot the heatmap results comparing the 35 most abundant genera between the control and 1000 ppm groups. When comparing the two groups, genera with a comparatively greater abundance in the corresponding samples are displayed in red, while those with relatively lower abundance are shown in blue (Figure 6). The heatmap results comparing the 1000 ppm and control groups revealed a slightly higher relative abundance at the phylum level of *Proteobacteria*, *Actinobacteria*, *Synergistetes*, *Thermomicrobia*, and *Spirochaetes* in the 1000 ppm group. In contrast, *Verrucomicrobia*, *Euryarchaeota*, *Saccaribacteria*, *Chloroflexi*, *Atribacteria*, and *Fusobacteria* were more abundant in the control group (Figure 6).

## 4. Discussion

Numerous alternatives for antibiotics are being investigated in experiments to determine their efficacy in both humans and animals. One alternative that is often utilized as a feed supplement in the poultry farming industry is probiotics, i.e., a non-antibiotic method with a number of positive impacts on the modulation of productive performance in poultry, including growth performance, egg performance, immunological response, nutrient digestion, and gut health [3,15,16,24,25].

As the results reveal, the inclusion of dietary *B. toyonensis* BCT-7112^T^ supplementation resulted in a remarkable enhancement in some measures of egg production in laying ducks. Suswoyo et al. [26] reported that Tegal laying ducks fed a probiotic mixture (*Lactobacillus* sp. and *Saccharomyces* sp.) showed the highest egg mass (*p* < 0.05). Based on a study in White layers conducted by Abdelqader et al. [23], the inclusion of *B. subtilis* PB6 at a dosage of 0.5–1.0 g/kg in the basal diet of hens resulted in significant improvements in both egg mass and egg weight. Egg mass also was significantly increased in hens fed a diet containing *B. subtilis* yb-114246 [27].

It is possible that the increased egg production could be a consequence of a boost in nutrient utilization [28], which is related to the positive effect of probiotics on the beneficial gut microbes [29] and gut architecture [30]. Furthermore, the potential mechanisms through which probiotics enhance these parameters can be ascribed to promoting dietary nutrient digestion and utilization through the upregulation of host digestive enzyme activities [31,32]. Consistent with our recent research, we observed that feeding *B. toyonensis* BCT-7112^T^ improved the gastrointestinal health of commercial meat ducks by modulating the beneficial bacteria associated with nutritional digestibility [3]. According to our findings, the beneficial impacts on egg production and average egg weight were caused by feeding *B. toyonensis* BCT-7112^T^. These occurrences could be attributed to improve nutrient absorption and metabolism, as probiotics have been shown to enhance gut microbiota composition and digestive enzyme activity, ultimately facilitating better nutrient utilization.

The lack of an effect on average daily feed intake, feed conversion ratio, and average daily egg production was similar to those of previous studies, e.g., Fathi et al. [33] in which *B. subtilis* did not affect egg production, feed intake, or FCR in laying hens. The dietary supplementation of *B. subtilis* TLRI 211-1 also did not alter the feed intake and egg production of Leghorn layers [34]. In a long-term experiment carried out by Guo et al. [35], it was shown that the inclusion of 10^5^ to 10^8^ CFU/kg *B. subtilis* CGMCC 1.921 in the diet of Hy-Line brown laying hens had no effect on feed intake or egg production rate. Adding *B. subtilis* ATCC PTA-6737 to the laying diet also had no statistically significant influence on FCR, daily feed intake, average egg weight, or laying rate [36]. Likewise, a feeding trial conducted on Pekin ducks also demonstrated that a dry direct-fed microbial diet containing *B. subtilis* (2.2 × 10^8^ CFU/g) had no significant impact on weight gain, feed intake, and FCR during the 1–21 day period [37]. Our study suggests that the absence of changes in the overall egg productivity could be due to the fact that, while probiotics enhance nutrient absorption, their influence on reproductive hormone regulation and follicle recruitment may be limited. Additionally, the production stage, breed characteristics, and environmental conditions may modulate the extent to which probiotics impact the laying performance.

In other studies, a combination of *B. subtilis* and *Clostridium butyricum* as dietary supplements resulted in substantial enhancements in daily feed intake, egg production rate, and body weight of laying ducks throughout the later stages of production [38]. The supplementation of Brown-Nick layers’ feed with a commercial probiotic (BioPlus 2B) increased egg production and FCR substantially (*p* < 0.05) [38]. Furthermore, the inclusion of *Clostridium butyricum* (5 × 10^4^ or 1 × 10^5^ CFU/g) in the diet improved egg performance of laying hens in the late-productive phase [39]. Clearly, data from the current study may differ from previous work due to factors such as the use of different bacterial strains, supplemental dose, environment, diet structure, animal models, breed, and age [33,40,41,42].

The lack of negative influence on all egg quality parameters, including yolk ratio, albumen ratio, eggshell ratio, albumen height, Haugh units, and yolk color, suggests that *B. toyonensis* BCT-7112^T^ can be used as a feed supplement in laying duck production at levels ranging from 200 to 1000 ppm without a negative impact on the overall egg qualities. Furthermore, the increased eggshell thickness due to feeding *B. toyonensis* BCT-7112^T^ supports data from previous studies with the dietary probiotic *B. subtilis* in laying hens [33], aged laying hens [43], and in broiler breeder hens [28,44], thus suggesting a similar effect of both probiotics. The results support data from previous studies indicating that adding *B. subtilis* to laying diets led to an increase in eggshell thickness and using the probiotic *B. subtilis* C-3102 as a feed additive in the basal diet improved eggshell thickness in aged laying hens. Also, supplementation with *B. subtilis* PB6 led to an increase in eggshell thickness in broiler breeder chickens. It is common knowledge that the eggshells of avian species consist primarily of calcium, along with various trace minerals. Increased calcium carbonate deposition in the eggshell can be linked to a pH reduction in the digestive system, which increases the solubility and digestibility of phosphorus and calcium [45]. Chang and Yu [46] noted that *Bacillus species* have the ability to generate short-chain fatty acids (SCFAs) by breaking down complex polysaccharides to modulate the gut microbiota composition due to decreased intestinal pH. Thus, it is possible that the enhancement in eggshell thickness in the current study was related to the reduction in intestinal pH caused by SCFA secretion during microbial fermentation, leading to an amplified calcium deposition on the shell glands.

However, while *B. subtilis* and *B. toyonensis* share common probiotic properties, their effects and underlying mechanisms can differ based on the host species and specific health parameters targeted. In order to have a complete understanding of these differences, further comparative research is necessary.

Decreasing NH_3_ emissions is a current challenge for poultry producers. Manure nitrogen content, temperature, pH, humidity, ventilation rate, and air velocity are the main factors determining NH_3_ volatility in poultry litter [47]. The production of NH_3_ in livestock housing systems is primarily attributed to the nitrogen (e.g., protein, urea, and uric acid) present in the undigested excreta from protein-rich feedstuffs [48]. Approximately 60–70% of the nitrogen present in poultry manure is excreted as uric acid and urea [49]. There have been worldwide attempts to mitigate NH_3_ emissions, employing adsorbent and acidifying additives [50,51], and decreasing the CP content in the diet [52]. Recent studies on the effects of nutrition on ammonia production in poultry have focused mainly on broilers and laying hens, but the influence of probiotics in ducks has rarely been investigated. In this context, probiotics can encourage the digestive activity of proteases and peptidases, promoting the absorption and transport of small peptides and amino acids through epithelial cells [53], and decreasing uric acid–nitrogen excretion [48]. Based on our results, including *B. toyonensis* BCT-7112^T^ could reduce the amount of NH_3_ emission released from the laying duck litter. The observed mechanism appears to be associated with enhanced protein digestibility and nitrogen utilization efficiency, consequently resulting in reduced excretion of nitrogenous waste products.

Recently, it has been reported that ammonia causes changes in the abundance of cecal microflora and a decline in the productive performance of laying ducks [5]. Vilela et al. [54] demonstrated that uric acid degradation by microbial uricase in excreta was the primary cause of NH_3_ synthesis. Similarly, Santoso et al. [55] observed that suppressing urease-producing microbiota by feeding a dried culture of *B. subtilis* significantly decreased NH_3_ concentration. Multi-strain probiotics (*B. subtilis, P. guilliermondii*, and *L. plantarum*) also reduced NH_3_ production by approximately 46% during in vitro gas fermentation using the cecal contents of laying hens [56]. Our study did not investigate gut microbial urease activity, nitrogen metabolism pathways, feed digestibility, or protein utilization. Future studies should conduct further analyses to understand the observed effects better.

The cecum of poultry is the primary location of fermentation due to the presence of a diverse microbial community, and it plays an essential function in the health and intestinal development of this species [57]. The cecal microbiome analysis was similar to previous studies, which indicated that Firmicutes, Proteobacteria, and Bacteroidetes are the predominant taxa in the duck cecum and ileum, comprising over 80% of the gut microbiota [3,58,59,60]. As evidenced by the OTU number of the intestinal microflora in the probiotic group in the current experiment being significantly higher than that of the control group, feeding probiotic *B. toyonensis* was advantageous in modulating the colonization and diversity of microbial communities. In terms of the diversity of microbial communities in the host gut, a moderate level of variety is beneficial for a stable gut microbiota. Not all gut microorganisms are useful: some are harmful, while others are conditioned. Some bacteria have little influence on the host, constituting the diversity and richness of the gut microflora [57].

In the current study, Bacteroides was among the most prevalent bacterial taxa in the *B. toyonensis* probiotic group. They are necessary for breaking down complicated compounds into smaller components for host growth [61]. They have been known for breaking down long-chain polysaccharides, and their metabolism can result in the production of acetate, propionate, or succinate, which actively improves the intestinal environment for beneficial microbes. Our findings show that supplementing *B. toyonensis* BCT-7112^T^ in laying duck diets did not significantly increase the proportions of the genera *Lactobacillus* and *Bacillus* in the gut. However, Bacteroides species can acidify nutritional broths in vitro almost as efficiently as *Bacillus and Lactobacillus* [62,63]. Both are known for producing organic acids, such as lactic acid, which play a crucial role in maintaining gut microbiome balance. The increase in these acid-producing bacteria can create an acidic environment in the gut, inhibiting the growth of pathogens and promoting digestive health [3,61,63]. Our result is comparable to that of Sun et al. [57], who observed that adding compound probiotics to duck diets increased the number of OTUs and the abundance of Bacteroidetes. We also observed that adding 1000 ppm of *B. toyonensis* BCT-7112^T^ to the diets decreased the amount of harmful bacteria, such as Fusobacterium. Kollarcikova et al. [64] reported that the cecal microbiota of poorly performing adult hens was characterized by a high abundance of Fusobacterium (around 20% of cecal microbiota). Moreover, other studies reported that Fusobacterium is a proinflammatory bacterium [65].

Our findings indicate that supplementing with *B. toyonensis* BCT-7112^T^ enhanced the relative abundance of beneficial bacteria while reducing the relative abundance of harmful bacteria. However, to properly understand the impact of probiotic *B. toyonensis* BCT-7112^T^ on the relationship of the gut microbiome, transcriptome, and metabolome in ducks, we recommend performing a shotgun metagenomic study and RNA-seq analysis using next-generation sequencing.

## 5. Conclusions

In conclusion, the supplementation of *Bacillus toyonensis* BCT-7112^T^ in laying duck diets at the different inclusion levels tested significantly increased the average egg weight, egg mass, and eggshell thickness and decreased ammonia emission in the litter. At 1000 ppm (1000 mg/kg), *Bacillus toyonensis,* BCT-7112^T^*,* significantly increased the egg mass, and the beneficial bacteria involved in nutrient digestibility were more predominant. These positive effects took place while pathogenic bacteria numbers decreased. Overall, *Bacillus toyonensis* BCT-7112^T^ has the potential to serve as an effective probiotic and may function as an alternative to antibiotic growth promoters (AGPs) in the production of laying ducks. In order to confirm the effects observed in this study, further studies should be performed on laying ducks using different scenarios (e.g., different duck breeds, breeding environments, and management modes).

## Figures and Tables

**Table 1:** Ingredients and calculated chemical composition of a basal diet (g/kg, as-fed basis unless stated otherwise).

Ingredients	g/kg, As-Fed Basis
Wheat grain	280.0
Casava chip	130.0
Wheat bran	125.5
Broken rice	42.0
Molasses	30.0
Rice bran oil	19.8
Rapeseed meal	40.0
Palm kernel meal	30.0
Soybean meal	97.0
Extruded soybean	40.0
Pork meal, 530 g/kg CP	44.0
Fish meal, 620 g/kg CP	31.0
Calcium carbonate	81.2
Salt	2.0
L-Lysine	1.0
DL-Methionine	2.0
Mycotoxin adsorbents	1.8
Pigment	0.2
Premix ^1^	2.5
Total	1000.0
Calculated Chemical Composition Analysis ^2^	
Metabolizable energy, kcal/kg	2850
Crude protein	180.0
Crude fiber	40.2
Calcium	35.1
Available phosphorus	4.3
Total lysine	9.6
Total methionine	4.0
Total methionine and cystine	7.2

^1^ Vitamin–mineral premix provided per kilogram of diet: vitamin A (trans-retinyl acetate), 12,000 IU; vitamin D3 (cholecalciferol), 3000 IU; vitamin E (all-rac-tocopherol-acetate), 12 mg; vitamin K3 (bisulfate menadione complex), 3.6 mg; vitamin B1, 1.4 mg; vitamin B2, 5.4 mg; vitamin B6, 4.2 mg; vitamin B12 (cyanocobalamin), 0.02 mg; nicotinic acid, 9 mg; pantothenic acid (D-calcium pantothenate), 9 mg; folic acid, 0.6 mg; biotin, 45 mg; choline chloride, 210 mg; selenium, 0.18 mg; cobalt, 0.3 mg; iodine, 1.08 mg; zinc, 60 mg; iron, 54 mg; manganese, 96 mg; copper, 12 mg; ^2^ The nutrient values were calculated based on the analyzed nutrient values according to NRC [22].

**Table 2:** Laying productivity and egg quality of laying ducks fed dietary *Bacillus toyonensis* BCT-7112^T^ from 32 to 44 weeks of age.

Item	Dietary *Bacillus toyonensis* BCT-7112^T^, ppm				SEM ^1^	*p*-Value
	Control *n* = 55	200 *n* = 55	500 *n* = 55	1000 *n* = 55		
Egg performance						
Average daily egg production, %	80.3	81.0	80.4	82.9	1.21	0.876
Average egg weight, g/egg	69.3 ^a^	70.3 ^b^	70.8 ^b^	71.1 ^b^	0.16	<0.001
Egg mass, g/h/d	55.7 ^a^	57.0 ^ab^	57.0 ^ab^	59.0 ^b^	0.40	0.031
Average daily feed intake, g/h/d	142.2	143.5	139.7	141.1	1.68	0.894
Feed conversion ratio	2.55	2.51	2.45	2.39	0.04	0.494
Egg quality						
Yolk ratio, %	33.0	33.3	32.5	32.5	0.10	0.400
Albumen ratio, %	56.1	55.8	56.6	56.6	0.11	0.150
Eggshell ratio, %	10.9	10.9	10.9	10.9	0.04	0.998
Eggshell thickness, μm	344.5 ^b^	353.4 ^a^	351.5 ^a^	352.1 ^a^	1.00	0.007
Albumen height, mm	5.1	5.1	5.0	5.1	0.05	0.937
Haugh units	62.0	62.2	61.4	62.7	0.43	0.779
Yolk color	14.7	14.8	14.8	14.7	0.02	0.243

^a,b^ Different superscripts letters in the same row indicate significant differences (*p* < 0.05); ^1^ SEM, standard error of the mean.

**Table 3:** Alpha diversity index statistics by sample.

Treatments	Observed Species	Shannon	Simpson	Chao1	ACE
Control	803.00	6.22	0.94	857.35	857.91
1000 ppm	1760.80	7.46	0.97	1894.17	1878.61
SEM	28.11	0.12	0.17	20.57	53.31
*p*-value	<0.01	0.02	0.13	<0.01	<0.01

ACE = abundance-based coverage estimator. Values are mean ± pooled SEM (*n* = 5).

## Data Availability

Data produced in this study are available from the corresponding. authors upon reasonable request.
